# Maternal obesity during pregnancy leads to adipose tissue ER stress in mice via miR-126-mediated reduction in Lunapark

**DOI:** 10.1007/s00125-020-05357-4

**Published:** 2021-01-27

**Authors:** Juliana de Almeida-Faria, Daniella E. Duque-Guimarães, Thomas P. Ong, Lucas C. Pantaleão, Asha A. Carpenter, Elena Loche, Laura C. Kusinski, Thomas J. Ashmore, Robin Antrobus, Martin Bushell, Denise S. Fernandez-Twinn, Susan E. Ozanne

**Affiliations:** 1 University of Cambridge Metabolic Research Laboratories and MRC Metabolic Diseases Unit, Wellcome Trust-MRC Institute of Metabolic Science, Addenbrooke’s Hospital, Cambridge, UK; 2 Obesity and Comorbidities Research Center, Faculty of Medical Sciences, State University of Campinas, São Paulo, Brazil; 3 Department of Food and Experimental Nutrition, Faculty of Pharmaceutical Sciences, University of São Paulo, Food Research Center, São Paulo, Brazil; 4 Cambridge Institute for Medical Research, University of Cambridge, Hills Road, Cambridge, UK; 5 Cancer Research UK (CRUK), Beatson Institute, Glasgow, UK

**Keywords:** ER stress, Glucose metabolism, Lunapark, Maternal obesity, miR-126-3p, Nutritional programming

## Abstract

**Aims/hypothesis:**

Levels of the microRNA (miRNA) miR-126-3p are programmed cell-autonomously in visceral adipose tissue of adult offspring born to obese female C57BL/6J mice. The spectrum of miR-126-3p targets and thus the consequences of its dysregulation for adipocyte metabolism are unknown. Therefore, the aim of the current study was to identify novel targets of miR-126-3p in vitro and then establish the outcomes of their dysregulation on adipocyte metabolism in vivo using a well-established maternal obesity mouse model.

**Methods:**

miR-126-3p overexpression in 3T3-L1 pre-adipocytes followed by pulsed stable isotope labelling by amino acids in culture (pSILAC) was performed to identify novel targets of the miRNA. Well-established bioinformatics algorithms and luciferase assays were then employed to confirm those that were direct targets of miR-126-3p. Selected knockdown experiments were performed in vitro to define the consequences of target dysregulation. Quantitative real-time PCR, immunoblotting, histology, euglycaemic–hyperinsulinaemic clamps and glucose tolerance tests were performed to determine the phenotypic and functional outcomes of maternal programmed miR-126-3p levels in offspring adipose tissue.

**Results:**

The proteomic approach confirmed the identity of known targets of miR-126-3p (including IRS-1) and identified Lunapark, an endoplasmic reticulum (ER) protein, as a novel one. We confirmed by luciferase assay that Lunapark was a direct target of miR-126-3p. Overexpression of miR-126-3p in vitro led to a reduction in Lunapark protein levels and increased *Perk* (also known as *Eif2ak3*) mRNA levels and small interference-RNA mediated knockdown of Lunapark led to increased *Xbp1*, spliced *Xbp1*, *Chop* (also known as *Ddit3*) and *Perk* mRNA levels and an ER stress transcriptional response in 3T3-L1 pre-adipocytes. Consistent with the results found in vitro, increased miR-126-3p expression in adipose tissue from adult mouse offspring born to obese dams was accompanied by decreased Lunapark and IRS-1 protein levels and increased markers of ER stress. At the whole-body level the animals displayed glucose intolerance.

**Conclusions/interpretation:**

Concurrently targeting IRS-1 and Lunapark, a nutritionally programmed increase in miR-126-3p causes adipose tissue insulin resistance and an ER stress response, both of which may contribute to impaired glucose tolerance. These findings provide a novel mechanism by which obesity during pregnancy leads to increased risk of type 2 diabetes in the offspring and therefore identify miR-126-3p as a potential therapeutic target.

**Graphical abstract:**

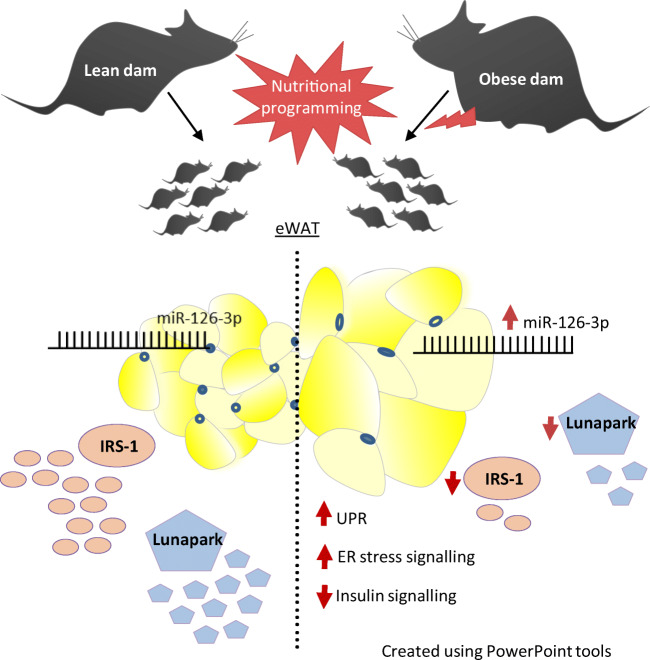

**Supplementary Information:**

The online version contains peer-reviewed but unedited supplementary material available at 10.1007/s00125-020-05357-4.

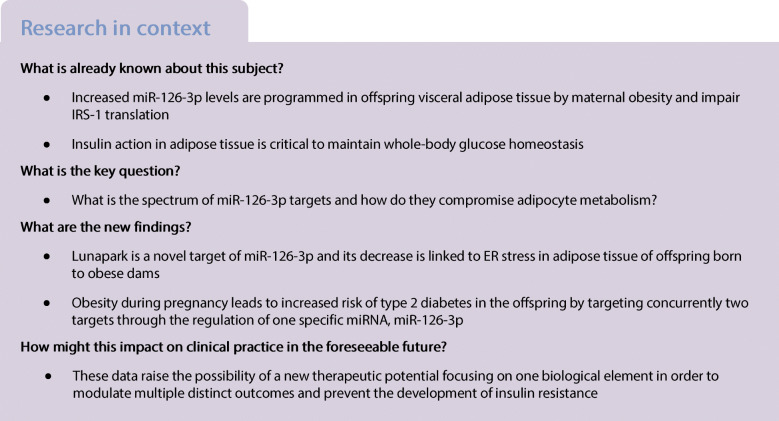



## Introduction

Overweight and obesity have reached epidemic proportions in both developed and developing countries [1]. Obesity is associated with an increased risk of a number of metabolic diseases including type 2 diabetes [2]. In part, this is thought to be the result of obesity-associated insulin resistance and activation of a variety of stress-related pathways in adipose tissue [3–7]. In addition to the well-established detrimental effects of obesity on the current health of an individual, there is accumulating evidence to suggest that obesity during pregnancy is particularly detrimental. This is because not only does obesity during pregnancy have an immediate detrimental impact on the mother (including increased risk of gestational diabetes and pregnancy-associated hypertension), it also has long-term programmed impacts on offspring health [8, 9] including increased risk of cardiometabolic disease [10]. This programming effect is encompassed by the developmental origins of health and disease (DOHaD) hypothesis [11].

The molecular mechanisms by which exposure to an obesogenic environment in utero mediates long-term effects on an individual’s health remain poorly understood. However, changes in the epigenome of cells have been proposed to play an important role [12]. This includes changes in DNA methylation, histone modifications and levels of small non-coding RNAs such as microRNAs (miRNAs) [13]. miRNAs regulate gene expression by specifically binding to the 3′ untranslated region (UTR) of mRNA-target transcripts and act to block its translation. The target sequence of one miRNA can be found in multiple mRNA transcripts thus leading to coordinated regulation of a network of genes [14]. A growing number of publications have reported dysregulation of miRNAs in various tissues following exposure to maternal obesity [15–18].

We showed previously that the expression of the miRNA miR-126-3p was increased in epididymal white adipose tissue (eWAT) of 8-week-old offspring of obese dams. This effect was cell-autonomous as it was retained following the in vitro culture and differentiation of programmed pre-adipocytes [15]. Since miRNAs generally regulate a gene network, the broader spectrum of miR-126-3p targets and the functional implications of their dysregulation has up till now remained unknown.

The aim of the current study was therefore to first take a proteomic approach to define targets of miR-126-3p in vitro and then to explore the long-term metabolic outcomes of their dysregulation as a consequence of maternal obesity.

## Methods

### Animal model and study design

This study was approved by the animal welfare and ethical review process of the University of Cambridge and was conducted according to the Home Office Animals (Scientific Procedures) Act 1986 following ethical review by the University of Cambridge Animal Welfare and Ethical Review Body (AWERB). From the third week of life, female C57BL/6J mice were fed ad libitum, either a balanced chow RM1 diet (7% simple sugars, 3% fat, 15% protein [wt/wt], 14.65 kJ/g, Special Dietary Services, Dietex International, Witham, UK) or a highly palatable energy-rich obesogenic diet (10% simple sugars, 20% animal fat, 23% protein [wt/wt], 18.84 kJ/g, Special Dietary Services) and sweetened condensed milk (55% simple sugars, 8% fat, 8% protein [wt/wt], 13.4 kJ/g, Nestle, UK) fortified with mineral and vitamin mix (AIN93G, Special Dietary Services). All details and specifications about the diet composition and the diet-induced maternal obesity model have been described previously [19]. Dams remained on their respective diets during pre-pregnancy, pregnancy and lactation periods. All females (fed chow or the obesogenic diet), were mated with chow-fed male mice. Dams were allowed to litter and the first litter was culled after weaning. This first pregnancy ensured the mice were proven breeders. After a week, mice were re-mated for a second pregnancy and day 1 of pregnancy was defined by the appearance of a post-copulatory plug. Offspring were standardised to 6 pups/litter 48 h after delivery by random selection by an animal technician not involved in any phenotypic analysis, to ensure similar milk availability. All experiments on offspring post weaning were conducted on male offspring only to avoid the confounding effects of sexual dimorphism. In the current study, one male mouse/litter was used in each experimental protocol developed so in all cases ‘*n*’ represents the number of litters studied. At 21 days of age, offspring born from control or obese dams were weaned onto a balanced chow diet, generating two offspring groups as follow: CC, where dams and offspring, post weaning, were fed standard chow diet; and OC, where the obesogenic diet was provided to dams during pregnancy and lactation followed by post-weaning standard chow diet to the offspring. All offspring remained on the abovementioned diet until 8 weeks of age (for euglycaemic–hyperinsulinaemic clamps; see below) or 6 months of age (all other measurements). At 6 months of age, following a 4 h fast, offspring were killed by raising CO_2_ concentration. Epididymal, retroperitoneal and intraperitoneal fat pads were dissected, weighed, snap frozen and stored at −80°C until analysis.

### Euglycaemic–hyperinsulinaemic clamp

Euglycaemic–hyperinsulinaemic clamps were performed as described previously [20]. Mice (8 weeks old) were fasted for 4 h and then anaesthetised with a cocktail of ventranquil: dormicum: fentanyl (1:2:10 in 3 units of water, 10 μl/g body weight, administered intraperitoneally, Janssen-Cilag, Tilburg, the Netherlands). Core body temperature of the animals was maintained at 37°C using a servo-controlled thermopad (Harvard Instruments, UK). A tail vein was catheterised and flushed with saline (154 mmol/l NaCl) to establish patency. d-^3^H-glucose was then infused continuously via the tail vein (0.006 MBq/min in PBS, 50 μl/h, iv, 370–740 GBq/mmol, Perkin Elmer, UK). Upon achieving steady state at 60 min (basal state, ≈5.5 h fasted), two blood samples (≤50 μl each) were taken 10 min apart from the tail. A bolus dose of insulin was then injected (3.3 mU, i.v., Actrapid, human insulin, Novo Nordisk, Denmark) followed by infusion at a rate of 0.09 mU/min. Blood glucose levels were monitored every 5 min for the first 20 min after insulin administration and then at 10 min intervals until the end of the protocol (≤5 μl per sample, Alphatrak, Zoetis, USA). A lowered reading of blood glucose concentration was detected around 5–10 min after starting the insulin infusion. At this point, a variable rate glucose infusion (12.5% wt/vol. PBS, Sigma, UK) was initiated and adjusted every 5–10 min thereafter to maintain blood glucose concentrations at mean basal fasted levels. At 50 min post insulin infusion, 2-deoxy-glucose (^14^C-2DG, specific activity: 9.25–13.0 GBq/mmol, Perkin Elmer, UK) was injected intravenously. By 70 min post insulin administration, blood glucose levels were clamped at basal concentrations. Three further blood samples (≤50 μl each) were collected from the tail at 10 min intervals. The mice were then killed by cervical dislocation and samples of vastus lateralis muscle, eWAT, heart and liver were collected for analysis of tissue ^14^C-2DG content. Rates of glucose utilisation (glucose infusion rate) and uptake of ^14^C-2DG into adipose tissue, vastus lateralis muscle and heart were calculated as described previously [20].

### Glucose tolerance test

Mice (6 months old) were fasted for 16 h (overnight) prior to glucose tolerance testing. Tail blood was collected for basal glucose evaluation (Alpha TRAK, Abbot Logistics, the Netherlands). Mice had glucose solution (1 g/kg) injected intraperitoneally and further blood glucose measurements were made at timed intervals. Calculations were plotted and analysed using GraphPad version 7.0 (Prism, GraphPad, La Jolla, USA).

### miRNA expression

Total RNA was extracted from 3T3-L1 cells and eWAT samples using the miRNeasy mini kit (Qiagen, UK). To quantify miR-126-3p, hsa-miR-126-3p Assay ID: 002228 was used (Applied Biosystems, UK). Standard curves were generated using serial dilution of the pooled cDNA samples and miR-126-3p expression was normalised to U6 small nuclear RNA (assay ID: 001973, Applied Biosystems, UK) for 3T3-L1 pre-adipocyte samples and to snoRNA202 for eWAT (assay ID: 001232, Applied Biosystems, UK). Both reference genes showed no differences between the groups.

### mRNA level detection

Quantitative PCR (qPCR) was used to determine the relative mRNA levels of all genes described in electronic supplementary material (ESM) Table 1 as described previously [15]. Cyclophilin was used as a reference gene since its expression did not differ between groups. Expression changes were measured according to the comparative cycle threshold method. Total RNA concentration and purity were evaluated spectrophotometrically using the NanoDrop ND-1000 (Thermo Scientific, UK). Samples were analysed in duplicate.

### Western blotting

3T3-L1 pre-adipocytes and eWAT samples were homogenised in commercial RIPA lysis and extraction buffer (cat. no. 89900, Thermo Scientific) and protein extraction and western blotting performed as described previously [15]. Each membrane was blocked in 5% non-fat milk powder added to 0.05% TBS/T (1 × TBS, 0.05% Tween 20) and further incubated overnight with the respective primary antibody (anti-IRS-1, 1:1000 dilution [cat no. 06-248 Upstate Biotechnology, Millipore, USA] and anti-Lunapark, 1:200 dilution [cat no. NBP1–80637, Novus Biologicals, USA]) solution. Following washing in TBS/T solution, membranes were incubated with horseradish peroxidase-conjugated anti-rabbit (1:10000) or anti-mouse (1:10000) antibody (Jackson Immuno Research, Stratech, UK). Antibody binding was detected using Super Signal West Pico Chemiluminescent substrate (Thermo Scientific) and an ImageQuant LAS 4000 machine and quantified using ImageQuant LAS 4000 software (GE Healthcare, UK). Coomassie Blue staining was used in all gels and anti-alpha-tubulin (cat no. 4074, Abcam, UK) antibody blotted on each membrane to confirm equal loading of proteins and equal transfer efficiency of samples.

### Adipocyte number and area

eWAT was fixed in 10% formalin, sectioned and stained with H&E and images generated using an AxioScan Slide Scanner (Z.1 version, ×20 lens; Zeiss, Cambridge, UK). The number and area of each cell unit were quantified using Image J (NIH, USA; version 1.47v) and 50 adipocytes/image were considered in all calculations. Each section represents an animal; 3–4 animals/group were evaluated in this protocol.

### Quantification of crown-like structures

eWAT sections (*n* = 3 per group) were stained by H&E, coverslipped and then imaged on an AxioScan Slide Scanner. Images of whole sections were then analysed using HALO analysis software (v3.0.311.201, Indica Labs, Corrales, NM, USA). Specifically, the DenseNet neural network algorithm of the HALO AI module was trained to selectively identify crown-like structures across random images in both groups. Entire sections were then analysed using the trained algorithm while blinded to the groupings.

### 3T3-L1 cell culture

Undifferentiated 3T3-L1 pre-adipocytes were thawed and plated to grow in a 25 cm^2^ culture flask in supplemented (10% FBS, 200 mmol/l l-glutamine and 0.5% penicillin/streptomycin)] high-glucose DMEM (all reagents from Thermo Scientific) until they reached around 70–80% confluence at 37 °C under 5% CO_2_. Cells were harvested using trypsin, centrifuged (200 *g* for 5 min) and re-suspended using a complete high-glucose DMEM medium (as described above).

### miR-126-3p mimic transfection followed by pSILAC, in-gel trypsin digestion and HPLC, MS analysis and data processing

Undifferentiated 3T3-L1 pre-adipocyte cells were transfected using a scrambled miR (labelled CTL in the figures, cat no. YM00479902, Qiagen) or commercial miR-126-3p mimic (cat no. YM00473217, Qiagen) (50 nmol/l final concentration in both cases). After 8 h, control and miR-126 mimic cells were labelled using stable isotope labelling by amino acids in culture (SILAC) DMEM (cat no. 88364, Thermo Scientific) supplemented with medium (cat no. 88437, Thermo Scientific) or heavy lysine (cat no. 88437, Thermo Scientific), respectively, for 24 h. Samples were resolved by SDS-PAGE using a 4–12% Bis-Tris gel (Novex, NuPAGE, Thermo Scientific) with lanes excised and cut into 12 approximately equal chunks. Samples were reduced, alkylated and digested in-gel using Lys-C with the resulting peptides eluted for LC-MSMS analysis. LC-MSMS data were acquired on a Q Exactive with an EASY spray source coupled to an RSLC3000 nano UPLC (Thermo Fisher Scientific, Germany). Peptides were resolved using a 50 cm PepMap column (Thermo Scientific) with a gradient rising to 40% solvent B (80% MeCN, 0.1% formic acid) by 45 or 57 min. MSMS data were acquired in a top 10 DDA fashion. Data were processed in Maxquant 1.5.2.8 using a Uniprot *Mus musculus* database (downloaded on 29 Jan 2016, 24,802 sequences). Carbamidomethyl cysteine was set as a fixed modification and acetyl protein N-terminus and oxidised methionine were set as variable modifications. The ‘Requant’ function was enabled. For detailed methodology, refer to [21]. TargetScan 7.1 was used for target prediction (http://www.targetscan.org/mmu_71/).

### 3T3-L1 cell protein and RNA extraction

After transfection and labelling of undifferentiated 3T3-L1 pre-adipocytes, the respective culture medium was removed and immediately replaced by 1 ml of ice-cold PBS and cells were mechanically harvested from the respective plates. Each sample was centrifuged at 16000 *g* for 10 min at 4°C, in a sterile falcon flask. Transfected/labelled 3T3-L1 cells were re-suspended in 3 ml ice-cold PBS and 1/3 volume transferred to three different Eppendorf tubes designated for protein (mass spectrometry and western blotting) or RNA extraction and pelleted by centrifugation.

### Plasmids and cell culture

For validation of *Lnpk* as a direct target of miR-126-3p, a luciferase reporter construct was generated by subcloning the mmu-*Lnpk* 3′UTR sequence downstream of the *luc2* gene on a pmiRGLO vector (Promega). For the detection of endoplasmic reticulum (ER)-related transcription factor activity, we developed a modified pGL4.20 luciferase reporter plasmid (Promega, USA) containing tandem repeats of the consensus mammalian ER stress response element (ERSE) and a minimal promoter upstream of the *luc2* gene (pGL4.20-2ERSEc34-minP). A pRL-SV40 (Promega) plasmid containing the *Rluc* gene was selected and used as a control for transfection efficiency. Cells were cultured in high-glucose DMEM supplemented with 10% FBS, 200 mmol/l l-glutamine and 0.5% penicillin/streptomycin in a 37°C, 5% CO_2_ environment.

### Dual-luciferase reporter gene assay for validation of *Lnpk* as miR-126-3p direct target

Hek293 cells were seeded in a 96-well plate at a density of 1 × 10^4^ cells/well. After 48 h, cells were co-transfected with 10 ng of pmiRGLO constructs and either 50 nmol/l miRCURY LNA mmu-miR-126b-3p mimic or 50 nmol/l negative control mimic (Qiagen), using Lipofectamine 3000 transfection reagent (Invitrogen). Cells were harvested and lysed after 24 h of transfection and both firefly and renilla luciferase activity was measured using a Dual-Glo Luciferase Assay System (Promega).

### Dual-luciferase reporter gene assay for ERSE activity

Host undifferentiated 3T3-L1 pre-adipocytes were seeded into 96-well plates (2.5 × 10^3^ cells/well) 24 h prior to transfection. In order to evaluate ERSE targeting transcription factor activity upon transient *Lnpk* knockdown, cells were co-transfected with 50 ng pGL4.20-2ESREc34-minP vector and either a scrambled negative control RNA mix (50 nmol/l) or a small interfering RNA (siRNA) targeting *Lnpk* mRNA (50 nmol/l), using 0.20 μl Lipofectamine 2000 per well for 24 h. In parallel, after 6 h of transfection, selected cells were treated with either 6 nmol/l thapsigargin or vehicle for 18 h. Cells were then washed with PBS and both firefly and renilla luciferase activity were measured using a Dual-Glo Luciferase Assay System (Promega) following the manufacturer’s protocol. Firefly luciferase activity in pGL4–20-2ESREc34-minP transfected cells (five replicates) was normalised by the renilla luciferase activity detected on the same extract, and the resulting ratio was later normalised by firefly:renilla luciferase activity.

### Statistical analysis

Data were analysed using Prism 6 (GraphPad, USA). With the exception of luciferase experiments, for the in vitro data ‘*n*’ corresponds to independent experiments that were developed in distinct cell line passages*.* For data regarding animal samples, ‘*n*’ refers to the number of litters evaluated, meaning that each male mouse analysed was born to a different dam. Glucose infusion rate during hyperglycaemic–euglycaemic clamp (Fig. 5b) and blood glucose measurements during glucose tolerance test (Fig. 6f) were analysed by two-way (repeated measures taken from the same animals) ANOVA followed by Bonferroni’s multiple comparisons test. All other data were analysed using unpaired Student’s *t* test*.* Data are presented as means ± SEM. For all statistical analysis, *p* < 0.05 was considered significant.

## Results

### Lunapark is a direct target of miR-126-3p

**Fig. 1:**
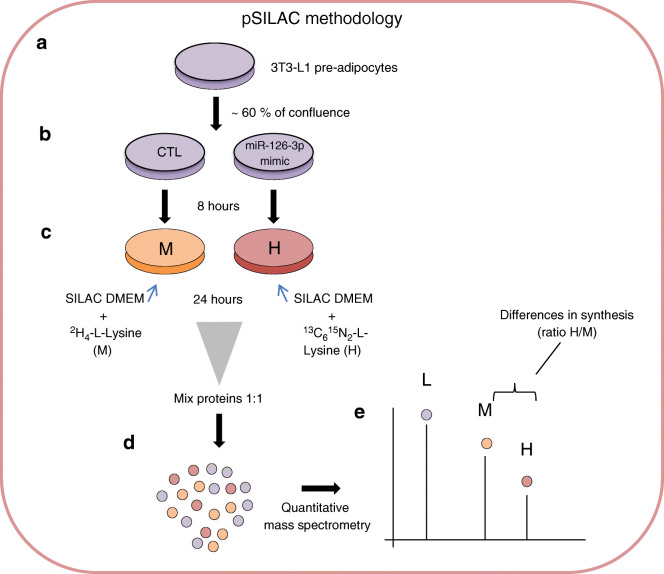
miR-126-3p overexpression followed by pSILAC in 3T3-L1 pre-adipocytes to determine the general proteomic profile. (**a**) 3T3-L1 pre-adipocytes were grown in supplemented DMEM high-glucose media until they reached 60–70% confluence. From this point, (**b**) cells were transfected with scrambled (CTL) or miR-126-3p mimic for 8 h. (**c**) Later, normal DMEM media was replaced with SILAC DMEM containing medium lysine (M; for CTL) or heavy lysine (H; for miR-126-3p overexpression) for 24 h, the time required for 3T3-L1 cell turnover and incorporation of the respective amino acid. (**d**) CTL or miR-126-3p-mimic-treated cell protein extracts were combined at the same proportion (based on protein quantification) and (**e**) differences in protein expression were detected by quantitative mass spectrometry

**Fig. 2:**
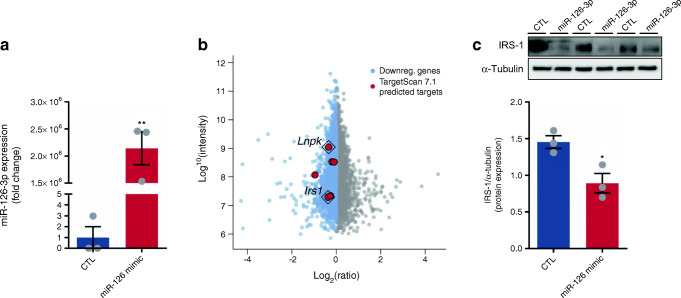
Protein general profile after miR-126-3p overexpression. (**a**) miR-126-3p expression normalised by U6 housekeeping mRNA, *n* = 3 per group. (**b**) Scatter plot representing the general proteomic profile found for miR-126-3p-mimic-transfected 3T3-L1 pre-adipocytes. The normalised heavy (miR-126-3p)/medium (scrambled; CTL) ratio was used as the basis of the graph. (**c**) IRS-1 protein expression normalised by α-tubulin, *n* = 3 per group. Data were analysed by unpaired Student’s *t* test (**a**, **c**, CTL vs miR-126 mimic); *p < 0.05 and **p < 0.01. All the graphs represent mean ± SEM. Downreg., downregulated

**Fig. 3:**
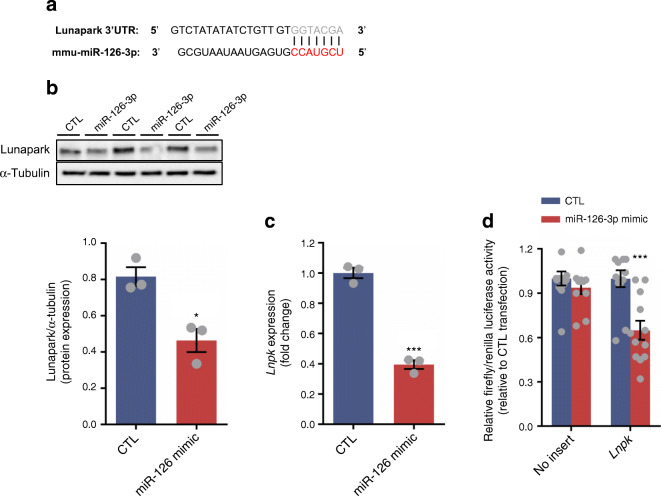
miR-126-3p directly regulates Lunapark protein expression. (**a**) Lunapark 3′UTR and its respective region of interaction within mmu-miR-126-3p sequence (miRNA seed sequence presented in red characters). (**b**) Representative blots and graphical representation of Lunapark protein expression normalised to α-tubulin, *n* = 3 per group. (**c**) *Lnpk* mRNA expression normalised by cyclophilin, *n* = 3 per group. (**d**) Relative firefly luciferase normalised by renilla luciferase activity measured in cells transfected with either a pmiRGlo vector with no insert or a pmiRGlo vector containing *Lnpk-3*’ UTR sequence, *n* = 12 per group. Data were analysed by unpaired Student’s *t* test (**b**, **c**, scrambled [CTL] vs miR-126 mimic; **d**, CTL vs miR-126 mimic, with or without siRNA targeting *Lnpk*); *p < 0.05 and ***p < 0.001. All the graphs represent mean ± SEM

In order to define the panel of proteins targeted by miR-126-3p, we transfected 3T3-L1 pre-adipocytes with either a fluorescent commercial scrambled miRNA or miR-126-3p mimic followed by 24 h of differential labelling with medium (^2^H_4_-l-lysine) or heavy (^13^C_6_^15^N_2_-l-lysine) lysine, respectively (Fig. 1). The length of labelling was defined after checking 3T3-L1 pre-adipocyte turnover (ESM Fig. 1a) and fluorescent control/mimic strand incorporation (ESM Fig. 1b). As expected, cells transfected with miR-126-3p mimic had substantially increased levels of the miRNA (Fig. 2a). The pSILAC approach identified 4569 proteins that were being actively translated (Fig. 2b). MiRNA overexpression is expected to lead to downregulation of its direct targets [22]. Following this premise, we used a validated miR-126-3p target, IRS-1 protein, as an internal positive control to confirm the efficiency of the transfection and labelling performance and the ability of the methodology to detect direct targets. We observed decreased IRS-1 protein expression which we confirmed by western blotting (Fig. 2c). In order to elucidate which pathways are affected by the in vitro miR-126-3p overexpression, we exported the proteomics data to the Kyoto Encyclopedia of Genes and Genomes (KEGG) database resource. KEGG integrates a variety of resources, including genomic and chemical data, to predict the cell routes/functions modulated in a specific context. An increase in miR-126-3p levels in 3T3-L1 pre-adipocytes modulated a number of different pathways, including both phosphoinositide 3-kinase (PI3K)–Akt and mammalian target of rapamycin (mTOR) signalling components (ESM Table 2). On the basis of the normalised heavy/medium peptide ratio found for IRS-1 (H/M = 0.773), a previously validated target [15], we defined 0.8 as the cut-off ratio and focused on proteins that were downregulated by a ratio value of 0.8 or lower. Using this cut-off, we identified 373 potential targets (ESM Table 3). This list includes those that are direct targets of miR-126-3p as well as those that are indirectly regulated by the direct targets. In order to identify those that were robust direct targets we screened the 3′UTR sequence of the potential targets and the miRNA seed sequence (using UCSC Genome Browser; genome.ucsc.edu). For maximum stringency, we designated as direct targets proteins that had at least six nucleotides matching the seed sequence of miR-126-3p. This stringent approach minimises risk of false positives but does not exclude completely that some potential targets were not included as candidates to follow up and formally to validate by luciferase assay. This strategy identified six potential direct targets of miR-126-3p (red dots, Fig. 2b). Of these, Lunapark demonstrated the greatest intensity and a notable downregulation in the pSILAC data (normalised H/M ratio = 0.786). Consistent with its dysregulation we found that the Lunapark 3′UTR sequence contained two complete miR-126-3p seed sequences (7 sequential nucleotides, Fig. 3a). This pSILAC data were validated by western blotting of Lunapark protein in 3T3-L1 pre-adipocytes overexpressing miR-126-3p (Fig. 3b). In addition, we also found a reduced level of *Lnpk* mRNA in these samples (Fig. 3c). We then confirmed that Lunapark was a direct target of miR-126-3p through use of a luciferase reporter assay. When the 3′UTR of Lunapark was inserted downstream of the luciferase gene in a reporter vector, transfection with miR-126-3p led to decreased luciferase activity in comparison with those transfected with a scrambled miRNA (Fig. 3d).

### Silencing of Lunapark affects ER components in 3T3-L1 pre-adipocytes

**Fig. 4:**
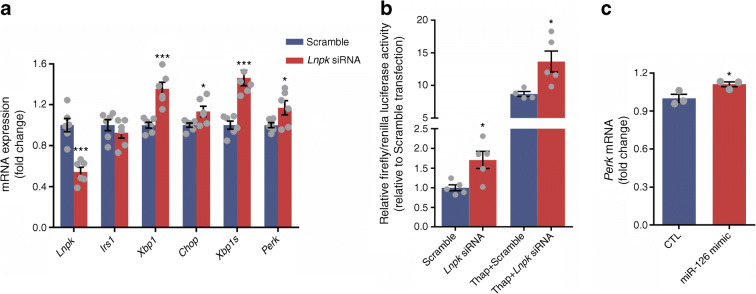
Lunapark inhibition activates ER stress response and miR-126-3p overexpression increases *Perk* mRNA expression in vitro. 3T3-L1 pre-adipocytes were transfected either with scrambled or *Lnpk* siRNA (**a**, **b**) or with scrambled (CTL) or miR-126-3p mimic (**c**) prior to the following measurements. (**a**) qPCR of *Lnpk*, *Irs1* and the altered ER-related genes, *n* = 5–6 per group. (**b**) ER stress response element targeting transcription factor activity measured through luciferase activity, *n* = 5 per group. (**c**) qPCR of *Perk* mRNA levels, *n* = 3. Data were analysed by unpaired Student’s *t* test (**a**, Scramble vs *Lnpk* siRNA; **b**, Scramble vs *Lnpk* siRNA, with or without thapsigargin; **c**, CTL vs miR-126 mimic); *p < 0.05 and ***p < 0.001. All the graphs represent mean ± SEM. Thap, thapsigargin

Lunapark is a conserved protein among different eukaryotic species and constitutes a key component of the three-way junctions of the peripheral ER network where its absence leads to losses of ER morphology/function [23]. Lunapark partial silencing, through use of an siRNA resulted in increased expression of a variety of ER stress-related components including *Xbp1*, *Chop* (also known as *Ddit3*), spliced *Xbp1* and *Perk* (also known as *Eif2ak3*) (Fig. 4a), a profile consistent with ER stress, despite similar *Irs1* mRNA levels. This effect of *Lnpk* knockdown was confirmed by evaluation of the ER stress presence directly using an ER stress response element reporter assay (Fig. 4b). *Lnpk* partial silencing resulted in an increase in luciferase activity indicating a positive stress response in comparison with the cells transfected with a scrambled siRNA. As expected thapsigargin, a potent apoptosis inducer, also led to an increase in luciferase activity compared with the control group. Together, thapsigargin and *Lnpk* silencing showed the most pronounced luciferase activity, indicating exacerbation of the stress response (Fig. 4b). Transfection with miR-126-3p mimic led to a similar increase in *Perk* (Fig. 4c) but no increase in *Xbp1*, spliced *Xbp1* or *Chop* (data not shown).

### Maternal obesity promotes long-term downregulation of IRS-1 and Lunapark through miR-126 programming in epididymal adipose tissue of adult offspring

**Fig. 5:**
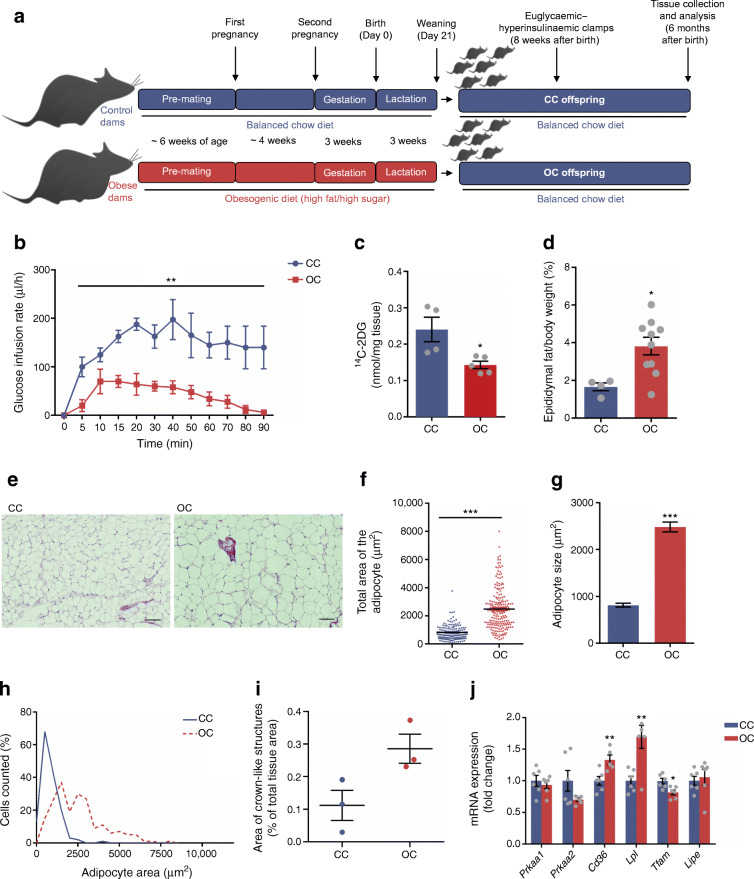
Maternal obesity compromises adipocyte morphology and functionality of adult offspring. (**a**) Schematic representation of the animal model used in the current study. (**b**) Euglycaemic–hyperinsulinaemic glucose infusion rate during clamp and (**c**) ^14^C-2DG uptake into adipose tissue of 8-week-old male offspring, *n* = 4–5 per group. (**d**) Relative epididymal fat of 6-month-old male offspring, *n* = 4–10 per group. (**e**) Representative H&E images (scale bars, 100 μm; *n* = 3–4), (**f**) total area, (**g**) size and (**h**) frequency distribution of adipocytes of CC and OC offspring, *n* = 3–4 animals per group, 50 adipocytes/animal. (**i**) Area of crown-like structures (*n* = 3 per group, *p* = 0.05) and (**j**) relative expression of genes related to lipid metabolism, *n* = 5–6 per group. Data in (**b**) were analysed by two-way (repeated measures) ANOVA followed by Bonferroni’s multiple comparisons test. Data in (**c**, **d**, **f**, **g**, **i**, **j**) were analysed by unpaired Student’s *t* test (CC vs OC offspring); *p < 0.05, **p < 0.01 and ***p < 0.001. All the graphs represent mean ± SEM

**Fig. 6:**
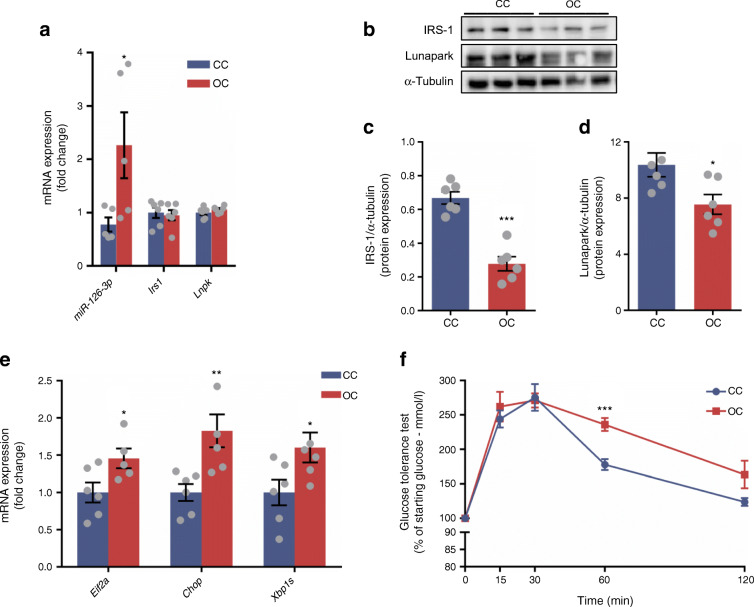
Maternal obesity compromises glucose and ER homeostasis concomitantly through miR-126-3p programming in eWAT of adult offspring. (**a**) Normalised relative miR-126-3p, *Irs1* and *Lnpk* expression (*n* = 5 per group). (**b**) Representative blots followed by normalised (**c**) IRS-1 and (**d**) Lunapark protein expression, *n* = 6 for both. (**e**) Altered relative expression of ER-related genes, *n* = 5–6 per group. (**f**) Percentage of starting glucose during glucose tolerance test, *n* = 6–7 per group. Data in (**a**, **c**–**e**) were analysed by unpaired Student’s *t* test (CC vs OC offspring). Data in (**f**) were analysed by two-way (repeated measures) ANOVA followed by Bonferroni’s multiple comparisons test; *p < 0.05, **p < 0.01 and ***p < 0.001. All the graphs represent mean ± SEM

We previously showed that increased miR-126-3p in adipose tissue is programmed cell-autonomously by maternal obesity in parallel with a reduction in IRS-1 protein in young adult (8-week-old) male offspring [15]. In the current study we aged some offspring of control and obese dams and studied them at 6 months of age (see schematic Fig. 5a). At 8 weeks of age euglycaemic–hyperinsulinaemic tests revealed that at this age offspring of obese dams required a lower glucose infusion rate, indicating whole-body insulin resistance (Fig. 5b) and that adipose tissue is a clear site of insulin resistance (Fig. 5c). At 6 months of age there remained no difference in body weight (33.94 g ± 1.58 CC vs 34.69 g ± 2.90 OC offspring) and there was no difference in fasting plasma insulin levels (238.6 pmol/l ± 79.33 CC vs 306.9 pmol/l ± 45.42 OC offspring), however OC offspring had increased eWAT mass (Fig. 5d). Retroperitoneal and intraperitoneal fat pads masses were, however, similar in CC and OC groups (ESM Fig. 2). Not only was the eWAT amount found to be increased in OC offspring but adipocyte morphology was also altered (Fig. 5e). Total area of adipocytes (Fig. 5f) and overall adipocyte size (Fig. 5g) were increased in OC compared with CC offspring and OC offspring had a higher number of larger adipocytes and a dramatic reduction in cells smaller than 2000 μm^2^ (Fig. 5h**)**. As the adipocyte diameter of OC offspring was positively regulated by maternal obesity, we also evaluated the area of crown-like structures (Fig. 5i) and observed an increase in the OC group. We measured the expression of genes involved in lipid metabolism [24–26] and among these, *Fat/cd36* (also known as *Cd36*) and *Lpl* were increased whereas *Tfam2* was decreased in eWAT of OC compared with CC offspring (Fig. 5j). We demonstrated that the programmed increase in miR-126-3p expression previously observed in 8-week-old offspring [15] was sustained in eWAT of 6-month-old male offspring (Fig. 6a), as was the reduction in IRS-1 protein (Fig. 6b, c). Furthermore, consistent with our in vitro findings, we observed that Lunapark (Fig. 6b, d) protein levels were also reduced in the adipose tissue from the offspring of obese dams. In both cases there was no change in the mRNA levels (Fig. 6a), suggesting that this reduction in vivo resulted from post-transcriptional regulation by miR-126-3p. Additionally, we observed increased *Eif2a*, *Chop* and spliced *Xbp1* in the eWAT of offspring of obese dams compared with controls (Fig. 6e), which supports a molecular phenotype associated with elevated ER stress. Consistent with these molecules being related to dysregulated metabolism, the offspring of obese dams at this age displayed a significant delay in glucose clearance compared with control animals during a glucose tolerance test (Fig. 6f).

## Discussion

We have shown previously that maternal obesity programmes epididymal fat of young male mice offspring to overexpress miR-126-3p, resulting in impairment of the insulin signalling pathway via direct downregulation of IRS-1 protein levels [15]. In the current study, we demonstrate that maternal obesity has lasting effects on miR-126-3p levels in eWAT of 6-month-old mice. We further demonstrated that at 8 weeks of age the offspring of obese dams had whole-body and adipose tissue insulin resistance. Since there is no difference in eWAT mass comparing CC and OC animals at this age, it is clear that the whole-body insulin resistance seen in the OC group is independent of changes in adiposity and is instead related to an impairment in adipose tissue insulin sensitivity. The subsequent adipocyte hypertrophy observed in OC offspring at 6 months of age could further exaggerate the insulin resistance.

We identified Lunapark as a novel direct target of miR-126-3p, which we validated in vitro and in vivo through different methodologies including pSILAC, qPCR, western blotting, luciferase assay and computational analysis. Lunapark is an evolutionarily conserved membrane protein that participates in ER-shaping through stabilisation of three-way junctions. The maintenance of characteristic ER morphology is a requisite for its proper physiological function [23] and therefore, as demonstrated in this study, its dysregulation leads to ER stress. We therefore show for the first time that programmed dysregulation of a single adipocyte miRNA can lead to both adipose tissue ER stress and insulin resistance, both of which contribute to the development of glucose intolerance in our mouse model.

It is well described that the accumulation of unfolded protein triggers ER stress and malfunction. To re-establish homeostasis, the cell contains a specialised machinery/system known as the unfolded protein response (UPR). Classically, low levels of ER stress-induced UPR in an acute context appear as an adaptive physiological mechanism required to restore ER homeostasis. Conversely, prolonged and intense ER stress-induced UPR activates mechanisms that culminate in programmed cell death [27]. Although Lunapark has previously been described as an important component of the ER [28], its role in modulating specific components of the ER stress pathway is shown in the current study. It is important to emphasise that, along with IRE1 and activating transcription factor 6 (ATF6), protein kinase R-like endoplasmic reticulum kinase (PERK) constitutes the set of transducers that can be activated during the UPR process. Each one is responsible for triggering a particular signalling pathway containing specific targets. Lunapark silencing through siRNA delivery was accompanied by increased *Perk, Xbp1,* spliced *Xbp1* and *Chop* mRNA expression. Although the IRE1 transducer was not increased, the concurrent elevation of its downstream targets *Xbp1* and spliced *Xbp1*, indicates a pro-apoptotic cellular response [29, 30]. In parallel, enhanced *Perk* levels, one of the aforementioned ER-related initiators, followed by an increase in *Chop* levels, may contribute to an exacerbated ER stress response [31]. Overexpression of miR-126-3p also resulted in an increase in *Perk* mRNA levels but not *Xbp1* or spliced *Xbp1*. This may be because of the more modest knockdown of Lunapark by the miR-126-3p mimic compared with the siRNA. Although *Perk* was not identified as one of the altered ER-related genes in vivo, *Chop* mRNA levels were increased. As a downstream protein, C/EBP homologous protein (CHOP) promotes apoptosis under situations of prolonged stress after adaptive UPR mechanisms failed to recover cell homeostasis [31].

It is important to highlight that it would be interesting to use differentiated adipocytes or alternatively create a stable cell line to perform proteomic experiments silencing Lunapark. Unfortunately, it is not technically possible to use pSILAC in differentiated and non-dividing cells as the methodology requires cells to undergo many rounds of cell division to incorporate sufficient label in a short window of time. Using differentiated cells in pSILAC would lead to insufficient labelling of newly synthesised proteins, affecting the quality of data generated. We understand that this is a limitation of our study. Label-free quantification (LFQ) of proteins would be an alternative approach to pSILAC that could be performed in differentiated adipocytes. However, it is known that LFQ has a lower sensitivity and is less quantitative compared with stable isotope approaches. Additional limitations of our study are that we only included male offspring and did not carry out molecular analysis of all fat depots. We recognise that growing evidence suggests that there are sexually dimorphic responses to a suboptimal early environment [32] and therefore it is important to establish in the future if similar effects on miR-126 are observed in female adipose tissue.

Mechanisms of metabolic programming remain poorly understood. Epigenetic processes and their triggers are thought to be important mediating components. Most studies in this field have focused on DNA methylation and few have addressed whether methylation changes are causative or indeed determined the functional consequences of the changes in methylation [13]. More limited data are available on the role of miRNAs. However, as shown in this study, small RNAs are important nodes of metabolic regulation that impact on a network of genes and therefore their dysregulation can have profound metabolic consequences. The relationships between obesity, insulin resistance and ER stress have been reported [33, 34], although the mechanisms involved in these associations are not yet fully understood. From these studies, accumulating evidence shows that ER stress normally triggers insulin resistance or worsens this condition. In the current study, our observations provide novel evidence for an influence of maternal obesity on the programming of miRNA-mediated ER stress activation in the eWAT that could contribute to programmed insulin resistance along with other programming mechanisms. As miR-126-3p directly targets IRS-1 and Lunapark, decreases their expression and compromises their function, the ER stress resulting from reduced Lunapark levels is likely to contribute to an exaggeration of the insulin resistance generated by downregulation of IRS-1. Although experimentally the presence of off-target effects is not uncommon, our combination of approaches using specific molecular strategies and computational algorithms decreases considerably the chance of reporting an off-target as an actual target. It is also important to highlight that luciferase reporter assays have been described as the gold standard method to validate direct targets of miRNAs and, combined with the other approaches, decreases (almost completely) an off-target recognition [35]. Additionally, it is possible that miR-126-3p could act through additional targets/mechanisms to alter glucose homeostasis and that of course other programming mechanisms are likely to contribute to the programmed glucose tolerance observed.

Here, despite similar body weights and consumption of a balanced post-weaning diet, offspring born to obese dams displayed pronounced changes in eWAT mass and morphology of its adipocytes. Together, these findings suggest that a programmed overexpression of miR-126-3p leads to both ER stress and insulin resistance concomitantly and therefore provide a novel link between two pathways known to contribute to the pathogenesis of type 2 diabetes. Additionally, as miR-126-3p presents a key role in regulation of both of these processes, its suppression in vivo would be a potential therapeutic avenue to explore.

In summary, our findings implicate a novel mechanism of nutritional programming by which maternal obesity can affect a network of metabolic signalling pathways in the offspring through regulation of one specific miRNA, miR-126-3p. It raises a new therapeutic potential focusing on one biological element in order to modulate multiple distinct outcomes. Furthermore, our findings suggest that there are broader implications than previously thought for programmed dysregulation of miRNAs.

## Supplementary Information

ESM (PDF 243 kb)

## Data Availability

All data generated or analysed during this study are included in this published article (and its supplementary information files).

## Abbreviations

^14^C-2DG: 2-Deoxy-glucose (labelled)
CC: Dams and offspring fed standard chow
ER: Endoplasmic reticulum
ERSE: ER stress response element
eWAT: Epididymal white adipose tissue
LFQ: Label-free quantification
miRNA: MicroRNA
OC: Dams fed obesogenic diet, offspring fed standard chow
pSILAC: Pulsed stable isotope labelling by amino acids in culture
qPCR: Quantitative PCR
siRNA: Small interfering RNA
UPR: Unfolded protein response
UTR: Untranslated region
