# Supplementary material for: Maternal obesity during pregnancy leads to adipose tissue ER stress in mice via miR-126-mediated reduction in Lunapark
Source: Diabetologia. 2021 Jan 27;64(4):890–902. doi: 10.1007/s00125-020-05357-4 (PMC7940301; doi:10.1007/s00125-020-05357-4)
Supplement: Supplementary file 1 — (PDF 243 kb) [file 125_2020_5357_MOESM1_ESM.pdf]

ESM Table 1. Primer Sequences

Gene name	Forward sequence (5' to 3')	Reverse Sequence (5' to 3')
Eif2alpha	ACGTGGCAGCCTTACACTAC	CTGACCAGGAAGGACACCAAT
Xbp1	CTGAGTCCGCAGCAGGTG	GGCAACAGTGTCAGAGTCCA
Chop	CATGTTGAAGATGAGCGGGTGG	ACCAGGTTCTCTCTCCTCAGGT
Spliced xbp1	AGCTTTTACGGGAGAAAACTCA	GCCTGCACCTGCTGCG
Perk	TCCCCTAGATCCCCTGAACTT	TTTCGAGCTGAGTGCTCTACA
Lnpk	TATCTGTAGGTGCTGCTGCC	AGTCACAGCTGAGAAAAGCG
Irs1	GGACATCACAGCAGCAGAATGAAGAC	CGTGAGGTCCTGGTTGTGAAT
Fat/cd36	TGCACTCTCTCATCGGACTTC	CGTGGCCCGGTTCTACTACTAAT
Lpl	ATCGGGCCCAGCAACATTAT	ACTCCTCCTCCATCCAGTTGA
Cyclophilin	GGATTCATGTGCCAGGGTGG	GTTTGGTCCAGCATTTGCCA
Tfam	GGGCCATCATTCGTCGGC	GACAAGACTGATAGACGAGGGGA
Prkaa1/Ampk alpha 1	AAATCCGCCGGGAGATTCAG	TGATGACCTGGTACAGTTTGATGA
Prkaa2/Ampk alpha 2	GGCAAAGTGAAGACTACCAGG	CGCCCATGTTTGCAGATGTAG
Hsl	CGCCAGGACTGGAAAGAATTG	GAACGCTGAGGCTTTGATCT

Genes and their correspondent forward and reverse sequences.

ESM Table 2.

Kegg analysis						
ID	Description	Gene ratio	Bg ratio	pvalue	p.adjust	qvalue
mmu04510	***Focal adhesion***	23/217	208/7783	1.33E-08	1.74E-06	1.44E-06
mmu04512	***ECM-receptor interaction***	15/217	88/7783	1.55E-08	1.74E-06	1.44E-06
mmu04151	***PI3K-Akt signaling pathway***	26/217	351/7783	4.42E-06	3.32E-04	1.44E-06
mmu04150	***mTOR signaling pathway***	10/217	61/7783	6.00E-06	3.37E-04	2.75E-04
mmu05205	***Proteoglycans in cancer***	18/217	205/7783	1.50E-05	6.77E-04	5.60E-04
mmu04974	***Protein digestion and absorption***	10/217	90/7783	1.88E-04	7.05E-03	5.84E-03
mmu05206	***MicroRNAs in cancer***	18/217	276/7783	6.72E-04	1.89E-02	1.57E-02
mmu04919	***Thyroid hormone signaling pathway***	10/217	117/7783	1.52E-03	3.81E-02	3.16E-02
mmu05214	***Glioma***	7/217	65/7783	2.10E-03	4.72E-02	3.91E-02

Main pathways affected by overexpression of miR-126-3p, including PI3K-Akt and mTOR signaling pathways.

ESM Table 3 - Downregulated proteins considering 0.8 as a cut-off point.

Downregulated proteins after tranfecting 3T3-L1 cells with miR-126-3p mimic	
Protein Name	Ratio H/M normalized
Collagen alpha-2(VI) chain	0.087928
Collagen alpha-2(VI) chain	0.11872
Short/branched chain specific acyl-CoA dehydrogenase, mitochondrial	0.158
Malectin	0.17419
Dystrobrevin alpha;Dystrobrevin beta	0.17813
Collagen alpha-2(IV) chain;Canstatin	0.23754
Lysine-specific demethylase 2A	0.25038
Arginine/serine-rich coiled-coil protein 2	0.26134
Protein canopy homolog 3	0.2671
Testis-expressed sequence 11 protein	0.26782
ATP-binding cassette sub-family B member 7, mitochondrial	0.3332
APC membrane recruitment protein 1	0.34107
Gap junction protein;Gap junction alpha-1 protein	0.35706
Multiple epidermal growth factor-like domains protein 10	0.36137
MAP/microtubule affinity-regulating kinase 3	0.37414
Sphingosine-1-phosphate lyase 1	0.37775
Outer dense fiber protein 2-like	0.37823
Keratin, type II cytoskeletal 2 oral	0.39849
Probable ATP-dependent RNA helicase DDX52	0.3986
Methyl-CpG-binding domain protein 2	0.39945
Transforming growth factor-beta receptor-associated protein 1	0.40149
Plexin-A1;Plexin-A2	0.40198
Phospholipase DDHD2	0.40511
Thioredoxin domain-containing protein 5	0.41024
Ribonuclease T2	0.41858
Tubulin-specific chaperone C	0.4197
NAD kinase (nadk- GTAC)	0.42491
Gamma-glutamylcyclotransferase	0.42609
Neurobeachin	0.42849
Rab-like protein 1	0.44857
Acetyl-coenzyme A transporter 1	0.45529
Retinal dehydrogenase 1	0.4625
Syndecan-3	0.46858
Adenylyltransferase and sulfurtransferase MOCS3;Molybdopterin-synthase adenylyltransferase;Molybdopterin-synthase sulfurtransferase	0.47064
Serine-protein kinase ATM	0.47939
Rho GTPase-activating protein 12	0.48014
Collagen alpha-1(VI) chain	0.48049
Copper-transporting ATPase 1	0.48717
Vascular cell adhesion protein 1	0.49965
E3 ubiquitin-protein ligase UBR2	0.50071
Oligophrenin-1	0.50323
Large neutral amino acids transporter small subunit 1	0.51398
Vacuolar protein sorting-associated protein 18 homolog	0.51894
Histone-lysine N-methyltransferase SETDB1	0.51955
Down syndrome critical region protein 3 homolog	0.52001
Aminopeptidase B	0.52086
Vesicle transport protein GOT1B	0.52214
Ubiquitin-like modifier-activating enzyme ATG7	0.52474

Poly(ADP-ribose) glycohydrolase ARH3	0.52594
Transmembrane 7 superfamily member 3	0.52603
Palladin	0.52732
Integrin-linked protein kinase	0.52858
Ras-related protein Rab-31	0.52925
E3 ubiquitin-protein ligase listerin	0.52943
Protein osteopotentia	0.52968
Unconventional myosin-VIIa	0.52987
Intron-binding protein aquarius	0.53232
cAMP-dependent protein kinase type I-alpha regulatory subunit	0.53497
Sarcosine dehydrogenase, mitochondrial	0.53516
Suppression of tumorigenicity 5 protein	0.5377
Regulatory-associated protein of mTOR	0.53771
Tyrosine-protein kinase Fer	0.54636
Eukaryotic translation elongation factor 1 epsilon-1	0.54846
N-myc-interactor	0.54924
Inhibitor of nuclear factor kappa-B kinase subunit beta	0.55023
Interleukin-1 receptor accessory protein	0.55072
Trafficking protein particle complex subunit 11	0.55192
Alpha-1,2-mannosyltransferase ALG9	0.55467
Poly(ADP-ribose) glycohydrolase	0.5556
Bromodomain adjacent to zinc finger domain protein 2A	0.55696
Serine/threonine-protein kinase Nek7	0.55793
Putative adenosylhomocysteinase 3	0.56154
Twinfilin-2	0.56241
CD151 antigen	0.56351
Thrombospondin-1	0.56498
Tubulin alpha-1A chain;Tubulin alpha-3 chain	0.56955
Protein phosphatase 1F	0.5723
Mitogen-activated protein kinase kinase kinase 7	0.57745
5-3 exoribonuclease 1	0.57925
Stonin-1	0.58006
CD166 antigen	0.5818
Dehydrogenase/reductase SDR family member 9	0.58351
Bromodomain and WD repeat-containing protein 3	0.58353
Ribonucleoside-diphosphate reductase subunit M2	0.58828
Peroxisomal membrane protein PEX14	0.5884
Target of Myb protein 1	0.58948
Oxidized low-density lipoprotein receptor 1;Oxidized low-density lipoprotein receptor 1, soluble form	0.5895
Myomegalin	0.59499
Thioredoxin domain-containing protein 12	0.59751
Neurofibromin	0.60011
Zinc finger SWIM domain-containing protein KIAA0913	0.60122
Huntingtin	0.60282
Sodium/hydrogen exchanger	0.60416
Proteasome subunit beta type-5	0.60532
Actin-related protein 2/3 complex subunit 4	0.60772
Abelson tyrosine-protein kinase 2	0.61042
Trinucleotide repeat-containing gene 6B protein	0.61173
Cytoplasmic polyadenylation element-binding protein 4	0.61665
Sodium-coupled neutral amino acid transporter 2	0.61683

E3 ubiquitin-protein ligase SHPRH	0.61769
Amidophosphoribosyltransferase	0.61834
Aprataxin	0.61902
Protein strawberry notch homolog 1	0.61912
Tyrosine-protein kinase CSK	0.62059
Protein bicaudal C homolog 1	0.62092
Ezrin	0.62172
Hemoglobin subunit beta-1	0.62308
Low-density lipoprotein receptor	0.625
CD9 antigen	0.62519
FERM, RhoGEF and pleckstrin domain-containing protein 2	0.62646
F-box only protein 30	0.62745
Acyl-coenzyme A thioesterase 1	0.62761
Tripeptidyl-peptidase 1	0.62903
Protein kinase C;Protein kinase C alpha type	0.63162
Cone cGMP-specific 3,5-cyclic phosphodiesterase subunit alpha	0.63193
Keratin, type II cytoskeletal 5	0.63285
Mannosyl-oligosaccharide glucosidase	0.6331
ATP-dependent RNA helicase A	0.63438
TRMT1-like protein	0.63512
Mitochondrial dicarboxylate carrier	0.63624
Serine/threonine-protein phosphatase 6 regulatory ankyrin repeat subunit E	0.63889
NEDD8	0.64065
Eukaryotic translation initiation factor 4 gamma 3	0.64071
E3 ubiquitin-protein ligase DZIP3	0.64196
Glucosamine 6-phosphate N-acetyltransferase	0.64323
MAGUK p55 subfamily member 7	0.64404
Double-stranded RNA-specific adenosine deaminase	0.64456
Vacuolar protein sorting-associated protein 53 homolog	0.64487
TBC1 domain family member 1	0.64602
Uncharacterized protein C14orf45 homolog	0.64706
E3 ubiquitin-protein ligase TRIM32	0.64797
Vacuolar protein sorting-associated protein 52 homolog	0.64828
ATP-dependent RNA helicase DDX54	0.64831
ATP-binding cassette sub-family B member 6, mitochondrial	0.64993
Rab5 GDP/GTP exchange factor	0.65166
Lethal(2) giant larvae protein homolog 1	0.6519
Protein sprouty homolog 4	0.65233
Phosphatidate phosphatase LPIN2	0.65309
Proteasome activator complex subunit 4	0.65441
Tensin-3	0.65454
Teashirt homolog 3	0.65541
Coiled-coil domain-containing protein lobo homolog	0.65591
Integrin alpha-V;Integrin alpha-V heavy chain;Integrin alpha-V light chain	0.65611
Amyloid-like protein 2	0.65772
Probable cation-transporting ATPase 13A1	0.65819
M-phase phosphoprotein 6	0.65928
Serine/threonine-protein kinase mTOR	0.65949
Integrator complex subunit 1	0.66009
Sodium-dependent phosphate transporter 2	0.66151
Cyclin-dependent kinase 6	0.66322

Platelet-derived growth factor receptor alpha	0.66427
Enhancer of mRNA-decapping protein 4	0.66472
Collagen alpha-1(V) chain	0.66574
1-phosphatidylinositol 4,5-bisphosphate phosphodiesterase delta-1	0.66681
Thioredoxin domain-containing protein 9	0.66727
Sodium/potassium-transporting ATPase subunit beta-3	0.66792
Phosphatidylinositol transfer protein beta isoform	0.66818
Protein SREK1IP1	0.67058
Glycerol-3-phosphate dehydrogenase, mitochondrial	0.67094
Putative tRNA pseudouridine synthase Pus10	0.67129
Interferon-induced 35 kDa protein homolog	0.67779
Carboxypeptidase Q	0.67801
Probable ATP-dependent RNA helicase DHX36	0.67864
Integrin alpha-1	0.68112
ATP-dependent RNA helicase DDX55	0.6832
Protein jagunal homolog 1	0.68399
Integral membrane protein 2C;CT-BRI3	0.68555
Probable palmitoyltransferase ZDHHC20	0.68591
Glutathione S-transferase A1;Glutathione S-transferase;Glutathione S-transferase A2	0.68662
Peroxisomal carnitine O-octanoyltransferase	0.68958
Plexin-D1	0.69154
39S ribosomal protein L9, mitochondrial	0.69322
Cytosolic acyl coenzyme A thioester hydrolase	0.69585
Phosphoribosylformylglycinamidine synthase	0.69612
Protein CLEC16A	0.69627
Ubiquitin/ISG15-conjugating enzyme E2 L6	0.69688
Syndecan-1;Syndecan	0.69756
Annexin A11	0.69938
SRSF protein kinase 2;SRSF protein kinase 2 N-terminal;SRSF protein kinase 2 C-termina	0.70053
Polycystin-2	0.70116
Serine/threonine-protein kinase TAO2	0.70247
tRNA (guanine-N(7)-)-methyltransferase subunit WDR4	0.70329
Transmembrane protein 168	0.70381
Elongation factor 1-alpha	0.70398
E3 ubiquitin-protein ligase HECTD1	0.70574
Alpha-parvin	0.70676
Non-histone chromosomal protein HMG-17	0.70695
Lysosomal-associated transmembrane protein 4A	0.70745
Protein TANC1	0.70841
Zinc transporter 6	0.70884
Asparagine--tRNA ligase, cytoplasmic	0.70983
39S ribosomal protein L16, mitochondrial	0.70988
Protein FAM208A	0.71035
N-acetyllactosaminide alpha-1,3-galactosyltransferase	0.71041
Formin-like protein 3	0.71081
Collagen alpha-2(I) chain	0.71084
Glucosamine-6-phosphate isomerase 1	0.71238
Zinc transporter 1	0.71266
Rho GTPase-activating protein 35	0.71285
Basement membrane-specific heparan sulfate proteoglycan core protein;Endorepellin;LG3 peptide	0.71351
Caspase-3;Caspase-3 subunit p17;Caspase-3 subunit p12	0.71364

Developmentally-regulated GTP-binding protein 2	0.71397
Platelet-activating factor acetylhydrolase IB subunit alpha	0.71441
Chromodomain-helicase-DNA-binding protein 8	0.7146
DNA-directed RNA polymerase I subunit RPA1;DNA-directed RNA polymerase	0.71498
Actin-related protein 2/3 complex subunit 1B	0.71532
Secernin-3	0.7161
Mitochondrial carrier homolog 2	0.71611
Ubiquitin-conjugating enzyme E2 W	0.7173
Proteasome assembly chaperone 2	0.71942
Folate transporter 1	0.71947
Lactoylglutathione lyase	0.72177
Dedicator of cytokinesis protein 7	0.72244
Non-histone chromosomal protein HMG-14	0.72341
SPRY domain-containing protein 4	0.72362
Transducin-like enhancer protein 1;Transducin-like enhancer protein 4;Transducin-like enhancer protein 3	0.72443
Sphingomyelin phosphodiesterase 4	0.72471
Uncharacterized protein C2orf78 homolog	0.72533
5-AMP-activated protein kinase subunit beta-1	0.72566
Protein FAM49B	0.72676
CTP synthase 1	0.72699
CTD small phosphatase-like protein	0.72733
Bcl-2 homologous antagonist/killer	0.72756
Histone-lysine N-methyltransferase SETDB1	0.72848
Ras and Rab interactor 1	0.72908
Uncharacterized protein C1orf198 homolog	0.72998
Ephrin type-B receptor 3	0.73133
MAP kinase-activated protein kinase 3	0.73224
Glucosamine--fructose-6-phosphate aminotransferase [isomerizing] 1	0.73282
Interferon-induced protein with tetratricopeptide repeats 3	0.73309
Probable global transcription activator SNF2L2	0.73316
Collagen alpha-1(XII) chain	0.73332
Fatty acid-binding protein, adipocyte	0.73351
Signal peptide peptidase-like 2B	0.73596
Thrombospondin-2	0.73662
Probable ATP-dependent RNA helicase DDX47	0.73692
NGFI-A-binding protein 1	0.73777
Tetraspanin-6	0.73967
Vacuolar protein sorting-associated protein 13C	0.74047
Cullin-7	0.74255
Transmembrane protein 176B	0.74299
MOB kinase activator 1B	0.74397
Uncharacterized protein KIAA0090	0.74432
FYVE, RhoGEF and PH domain-containing protein 3	0.74453
PRA1 family protein 3	0.74522
Fermitin family homolog 2	0.74619
Protein-associating with the carboxyl-terminal domain of ezrin	0.74634
Importin subunit alpha-7;Importin subunit alpha	0.74652
Pre-mRNA branch site protein p14	0.7471
Centromere protein J	0.74806
Fos-related antigen 2	0.74811
GDP-L-fucose synthase	0.74826

Filamin-A	0.74831
Insulin-like growth factor 2 mRNA-binding protein 2	0.7485
UTP--glucose-1-phosphate uridylyltransferase	0.74873
Polypyrimidine tract-binding protein 3	0.74984
Activator of 90 kDa heat shock protein ATPase homolog 1	0.75056
Mannosyl-oligosaccharide 1,2-alpha-mannosidase IB	0.75096
Unconventional myosin-IXa	0.75246
Methionine--tRNA ligase, cytoplasmic	0.7527
Zinc finger protein 609	0.75323
Poly [ADP-ribose] polymerase 14	0.75358
Aldehyde dehydrogenase, cytosolic 1	0.75408
Transmembrane protein 185B	0.75437
AP-3 complex subunit delta-1	0.75465
Cleavage and polyadenylation specificity factor subunit 5	0.75616
Myotubularin-related protein 3	0.75778
Tripartite motif-containing protein 16	0.75898
Protein YIPF5	0.759
Probable E3 ubiquitin-protein ligase DTX2	0.75983
Protein FAM101B	0.75998
Armadillo repeat-containing X-linked protein 1	0.76013
Quinone oxidoreductase-like protein 1	0.76038
C-Jun-amino-terminal kinase-interacting protein 4	0.7608
ADP-ribosylation factor-binding protein GGA3	0.76083
Pantothenate kinase 4	0.76101
Astrotactin-2	0.76177
CMP-sialic acid transporter	0.76195
Kelch domain-containing protein 4	0.76201
Magnesium transporter protein 1	0.76241
Choline transporter-like protein 1	0.7646
Uncharacterized protein C6orf47 homolog	0.76464
Nuclear pore complex protein Nup133	0.76485
MAPK-interacting and spindle-stabilizing protein-like	0.76491
Dual specificity mitogen-activated protein kinase kinase 1	0.76548
WD repeat-containing protein 92	0.76551
1-acyl-sn-glycerol-3-phosphate acyltransferase delta	0.76562
TSC22 domain family protein 3	0.76578
DnaJ homolog subfamily C member 24	0.76771
Cytospin-B	0.76817
Signal transducer and activator of transcription 5B	0.76825
Uncharacterized protein KIAA0754	0.76847
Beta-parvin	0.76849
Ephrin type-A receptor 2	0.76858
PHD finger protein 10	0.76886
Target of EGR1 protein 1	0.7703
RNA polymerase II elongation factor ELL	0.77073
Mediator of RNA polymerase II transcription subunit 13-like	0.77112
LisH domain and HEAT repeat-containing protein KIAA1468	0.77135
Myosin-14	0.77143
Breast carcinoma-amplified sequence 3 homolog	0.77152
PRELI domain-containing protein 1, mitochondrial	0.77161
Dolichyl-diphosphooligosaccharide--protein glycosyltransferase subunit 2	0.77171

Cystatin-B	0.77217
CD99 antigen-like protein 2	0.7723
Solute carrier family 23 member 2	0.77302
UPF0767 protein C1orf212 homolog	0.77323
Insulin receptor substrate 1	0.77351
CAAX prenyl protease 1 homolog	0.77357
Adenylate cyclase type 9	0.77578
ERO1-like protein alpha	0.77586
1-phosphatidylinositol 4,5-bisphosphate phosphodiesterase gamma-1	0.77724
Amyloid beta A4 protein;N-APP;Soluble APP-alpha;Soluble APP-beta;C99;Beta-amyloid protein 42;Beta-amyloid protein 40;C83;P3(42);P3(40);	0.77753
Tuberin	0.77771
Palmitoyltransferase ZDHHC13	0.778
Eukaryotic translation initiation factor 3 subunit M	0.77867
Importin-13	0.77873
Proteasome-associated protein ECM29 homolog	0.77891
Lysocardiolipin acyltransferase 1	0.7795
Zinc finger homeobox protein 3	0.77957
Rho guanine nucleotide exchange factor 10	0.78016
Neutral amino acid transporter A	0.7818
Nuclear receptor coactivator 5	0.78188
Elongation factor 1-alpha 1;Elongation factor 1-alpha	0.78203
Kinesin-like protein KIF3B	0.78234
Rho GTPase-activating protein 1	0.78276
Small subunit processome component 20 homolog	0.78279
Meiosis arrest female protein 1	0.78298
Unconventional myosin-IXb	0.7833
Carnitine O-palmitoyltransferase 1, liver isoform	0.7834
Unconventional myosin-Ic	0.78369
3-hydroxy-3-methylglutaryl-coenzyme A reductase	0.7839
Protein C8orf37 homolog	0.78449
Probable methyltransferase-like protein 15	0.7849
Protein fat-free homolog	0.78512
Guanosine-3,5-bis(diphosphate) 3-pyrophosphohydrolase MESH1	0.78519
Zinc finger homeobox protein 4	0.78528
Rapamycin-insensitive companion of mTOR	0.78546
Replication protein A 70 kDa DNA-binding subunit	0.78585
Axin-1	0.78619
Fatty acid desaturase 3	0.78639
Protein KIAA0664	0.78668
Coatomer subunit alpha;Xenin;Proxenin	0.78671
Protein lunapark	0.78695
Collagen alpha-1(I) chain	0.78841
Anoctamin-10	0.78878
Tumor necrosis factor alpha-induced protein 2	0.78905
AP-2 complex subunit sigma	0.7895
Glycogenin-1	0.79056
Aspartyl aminopeptidase	0.79159
Ephrin type-B receptor 2	0.79183
Delta(3,5)-Delta(2,4)-dienoyl-CoA isomerase, mitochondrial	0.79188
Actin, aortic smooth muscle;Actin, gamma-enteric smooth muscle;Actin, alpha cardiac muscle 1;Actin, alpha skeletal muscle	0.79244
Eukaryotic translation initiation factor 4E type 2	0.79284

AP-3 complex subunit beta-1	0.7935
PAB-dependent poly(A)-specific ribonuclease subunit 2	0.7942
Nucleolar MIF4G domain-containing protein 1	0.79437
Coatomer subunit beta	0.79462
28S ribosomal protein S2, mitochondrial	0.79467
UPF0711 protein C18orf21 homolog	0.79526
28S ribosomal protein S5, mitochondrial	0.79551
Rho-associated protein kinase 1	0.79578
Dysferlin	0.79629
Kinesin-like protein KIF16B	0.7965
DNA damage-binding protein 1	0.79674
Peripheral plasma membrane protein CASK	0.79738
Beta-enolase;Enolase	0.79772
Dipeptidyl peptidase 9	0.79796
Pseudopodium-enriched atypical kinase 1	0.798
Importin-11	0.79805
Integral membrane protein 2B;BRI2, membrane form;BRI2 intracellular domain;BRI2C, soluble form;Bri23 peptide	0.79851
Biglycan	0.79946
CAP-Gly domain-containing linker protein 2	0.79957

ESM Fig. 1

a

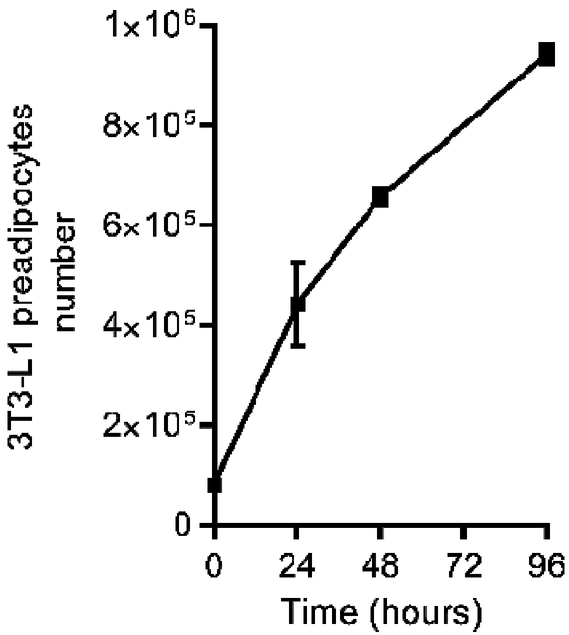

b

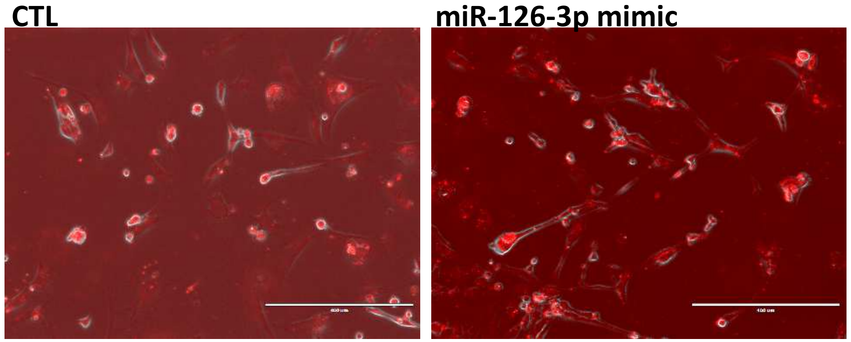

(a) Cell line turnover was stablished before miRNA mimic transfection and labelling protocols, n=4 per time point. (b) Representative images of fluorescent mock (Left) or miR-126-3p mimic (Right) transfected 3T3-L1 cells at x10 magnification, taken at 24h after transfection. Scale bars, 400 μm.

ESM Fig. 2

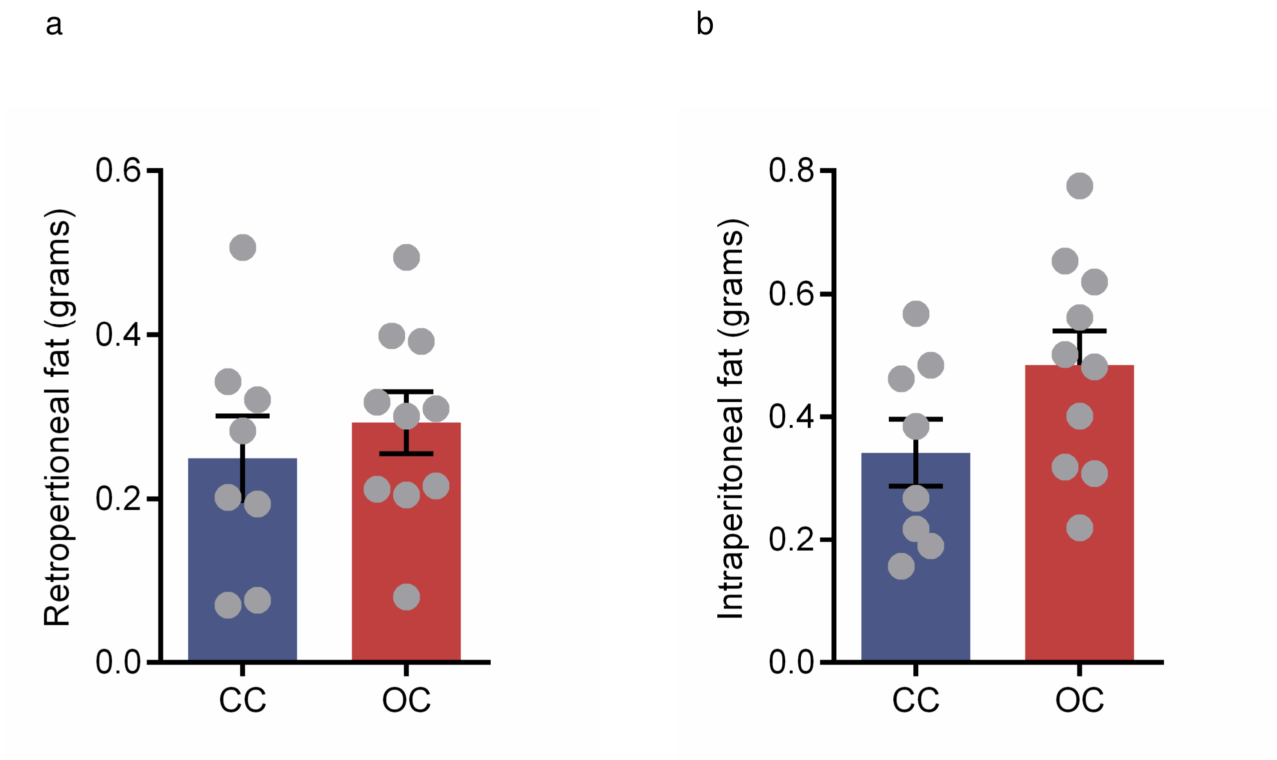

Absolute (a) Retroperitoneal and (b) Intraperitoneal fat depots weights of 6 months old male offspring, n=8-11 per group.
